# Effects of Two Ecological Governance Measures for Photovoltaic Power Stations on Plant Growth and Soil Nutrients

**DOI:** 10.3390/plants14050797

**Published:** 2025-03-04

**Authors:** Yajing Liu, Jingbo Zhang, Ren Mu, Danyang Wang, Zhaoming Wang, Jingyuan An, Xinle Li

**Affiliations:** 1 Experimental Center of Desert Forestry, Chinese Academy of Forestry, Dengkou 015200, China; 2 National Center of Pratacultural Technology Innovation (Under Way), Hohhot 011517, China

**Keywords:** photovoltaic power station, ecological governance, plant growth, soil nutrients

## Abstract

Ecological governance is essential to promoting the sustainable development of photovoltaic power stations in sandy regions and serves as a necessary measure for photovoltaic sand control. This study aims to investigate the impact of ecological governance measures on soil nutrients and plant growth, providing a theoretical foundation and scientific guidance for optimizing ecological management strategies in photovoltaic power stations located in sandy areas. The research focuses on two ecological governance measures: (1) the direct planting of *Haloxylon ammodendron* in bare sand in front of, between, and behind photovoltaic panels and (2) the planting of *H. ammodendron* after laying straw checkerboard barriers. The effects of these two measures on plant growth and soil nutrients were compared and analyzed across different positions relative to the photovoltaic panels (in front, between, and behind). The study revealed that the plant height of *H. ammodendron* was significantly higher than the control under both ecological governance measures in all three positions (*p* < 0.05). Furthermore, the crown width, aboveground biomass, underground biomass, and total biomass of *H. ammodendron* planted directly in bare sand as an ecological governance measure were significantly greater than those of plants grown after the installation of straw checkerboard barriers. A two-factor analysis of variance indicated that sampling location, ecological governance measures, and their interaction significantly affected the plant height and crown width of *H. ammodendron* (*p* < 0.01). Redundancy analysis demonstrated that soil available nitrogen was positively correlated with aboveground dry weight, underground dry weight, total biomass, and crown width, with all parameters increasing as soil available nitrogen content increased. Additionally, soil available nitrogen and soil available potassium were identified as key factors driving the growth of *H. ammodendron*. In conclusion, the ecological governance measures of planting *H. ammodendron* directly in bare sand within the photovoltaic park demonstrated superior growth and biomass outcomes compared with planting the species after the installation of straw checkerboard barriers.

## 1. Introduction

Deserts represent a critical component of terrestrial ecosystems, and China is among the countries most severely affected by desertification globally [1]. The extensive area and wide distribution of desertification significantly hinder the coordinated development of population, resources, and the environment in affected regions [2]. Desert regions experience scarce rainfall, severe wind erosion, sparse surface vegetation, and heightened sensitivity to human activities in response to climate change. Consequently, the risk of ecological “reverse” succession is exceptionally high [3].

Following China’s announcement of its “carbon peaking” and “carbon neutrality” goals [4], China has actively pursued sustainable and pollution-free clean energy to replace traditional fossil fuels, with photovoltaic power generation emerging as a key driver in achieving the “dual carbon” goals due to its low-carbon, high-efficiency, and sustainable advantages [5]. The construction of photovoltaic power plants requires extensive land and abundant solar energy. Deserts and Gobi regions, characterized by long sunshine hours, high light intensity, strong irradiance, and ample solar energy, provide ideal conditions for establishing photovoltaic power stations [6]. In 2021, President Xi Jinping proposed that China would continue to promote adjustments to its industrial and energy structures, vigorously develop renewable energy, and accelerate the planning and construction of large-scale wind and photovoltaic base projects in arid regions, the Gobi Desert, and desert areas [4,7]. The establishment of photovoltaic power stations has altered the local surface energy distribution and microclimate environment, influencing the ecological geochemical behavior of biological elements within the ecosystem, as well as associated material, energy, chemical, and biological processes [8]. This further influences soil function regulation and vegetation restoration [9].

Upon the completion of a photovoltaic power station, various ecological control measures, including engineering, biological, and chemical approaches, are typically implemented to achieve functions such as windbreak and sand fixation, water conservation, and soil erosion prevention and control. Studies have demonstrated that the ecological function of sand prevention and control of photovoltaic supports per unit area is greater than that of sand fixation forests and mechanical sand barriers of the same area [10,11]. Moreover, photovoltaic panels provide shading and rainwater collection, which can promote plant growth. As a result, plants growing beneath photovoltaic panels exhibit not only greater height and density but also significantly higher plant diversity and species richness compared with those outside the power plant [12]. Plants and soil are crucial components of ecosystems, and their dynamic relationship is interdependent [13]. Soil quality influences the growth, development, and distribution of aboveground plants, which, in turn, affects net primary productivity and the ecological functions of ecosystems [14]. Photovoltaic applications on land may not improve the chemical composition of soil in the short term, but they can affect soil temperature and moisture content. Tong et al. [15] conducted a study by planting *Astragalus laxmannii* at various locations around photovoltaic panels in the Kubuqi Desert and found that *A. laxmannii* within the photovoltaic power station exhibited excellent water retention performance near the periphery of the panels. In shaded areas of photovoltaic power plants, soil pH, conductivity, and available phosphorus and potassium content decreased compared with unshaded areas, while the soil bulk density increased [16]. Soil, as a medium for plant growth and development, indirectly reflects vegetation growth and provides essential nutrients for plants [17]. Plant growth improves soil structure and fertility through litter, root traits, secretions, and other processes, thereby promoting soil development, controlling erosion, and facilitating soil and water conservation [18,19]. The soil moisture content, organic matter content, and total nitrogen content in photovoltaic power stations located in high-altitude desert grasslands have increased compared with areas without photovoltaic power stations [20]. He et al. [21] found that the contents of soil organic carbon, mineral-bound organic carbon, and particulate organic carbon in the vegetation restoration area was significantly higher than in the shifting sand area, based on their study of vegetation restoration’s impact on soil organic carbon at the southern edge of the Tengger Desert. Additionally, the plant type is an important factor influencing nutrient content in photovoltaic land. Research has shown that planting *Glycyrrhiza uralensis* in the 0–30 cm soil layer of photovoltaic power plants results in higher organic matter content compared with planting *Leymus chinensis* and *Hedysarum scoparium* [22].

After the construction of photovoltaic power stations in sandy areas, shrub species such as *Haloxylon ammodendron*, *Caragana korshinskii*, and *Atriplex canescens*, which are well suited for growth in photovoltaic parks, were introduced to mitigate wind erosion and protect the photovoltaic panels and foundation piles. This ecological restoration and governance measures aimed to enhance the stability and sustainability of photovoltaic facilities. However, limited research has been conducted on the interaction between plants and soil at different locations under various patterns of ecological governance within photovoltaic power stations. Therefore, this study focuses on the 100 MW photovoltaic sand control park in Dengkou County, established by the Inner Mongolia State Power Investment Group and Yishite Group, to analyze the responses and changes in plant growth and soil nutrients at different locations between rows of photovoltaic panels under two ecological governance measures. The findings aim to provide a scientific basis for vegetation selection and the optimization of ecological environment protection for photovoltaic power stations in desert areas.

## 2. Results and Analysis

### 2.1. The Impact of Different Ecological Governance Measures for Photovoltaic Panels on the Growth of H. ammodendron

#### 2.1.1. Plant Growth Between Rows of Photovoltaic Panels

Under both ecological governance measures, the plant height of *Haloxylon ammodendron* was significantly higher than that of the control group (*p* < 0.05). Specifically, the plant height of LS-PF and CS-PF increased significantly by 48.12% and 42.47%, respectively, compared with the control (*p* < 0.05) (Abbreviations are used in different locations and ecological governance methods in the manuscript, with specific meanings as shown in Table 1). Additionally, the plant height of CS-PM increased significantly by 50.24% relative to the control (*p* < 0.05) (Table 2). The plant height of LS-PF *H. ammodendron* was significantly greater than that of CS-PF (*p* < 0.05). Additionally, the plant height of CS-PM and CS-PB was significantly higher than that of LS-PM and LS-PB (*p* < 0.05). The crown width of the *H. ammodendron* control group was significantly greater than that of LS-PF and CS-PF, with increases of 3.62% and 5.10%, respectively (*p* < 0.05). Furthermore, the crown width of LS-PM and LS-PB *H. ammodendron* was significantly larger than that of CS-PM and CS-PB (*p* < 0.05). A two-factor analysis of variance on the sampling location and treatment (ecological governance measures) revealed that the sampling location, ecological governance measures, and their interaction all had significant effects on the plant height and crown width of *H. ammodendron* (*p* < 0.01).

**Table 1:** The description of abbreviated nouns in the article.

Abbreviation		Description
Treatments	CK: control group	Control *Haloxylon ammodendron* was planted at the same time as the treatment group, without photovoltaic panel obstruction.
	LS: *Haloxylon ammodendron* planted on bare sandy land	The first ecological governance measure (treatment group 1) is to carry out ecological governance within the photovoltaic park after establishing a photovoltaic power station in desert areas. This treatment involves directly planting *Haloxylon ammodendron* in the exposed sandy area between the photovoltaic panels and the photovoltaic panel mechanical working road.
	CS: laying straw checkerboard barriers and planting *Haloxylon ammodendron*	The second ecological governance measure (treatment group 2) is to carry out ecological governance within the photovoltaic park after the establishment of the photovoltaic power station. This treatment involves laying straw checkerboard barriers on the exposed sandy land between the photovoltaic panels and the photovoltaic panel mechanical working road and then planting *Haloxylon ammodendron*.
Positions	PF: front eaves of photovoltaic panels	The area located 1 m away from the front eaves of the photovoltaic panels (as indicated in Figure 1), which receives sunlight from 8 am to 5 pm throughout the day, is referred to as the full-light zone.
	PM: between photovoltaic panels	The area located between the photovoltaic panels and the exposed sandy road (as shown in Figure 1), which receives sunlight from 11 am to 4 pm, is referred to as the semi-shaded and semi-sunny area.
	PB: behind photovoltaic panels	The area located 1 m behind the eaves of the previous photovoltaic panel (as indicated in Figure 1) cannot receive sunlight from 8 am to 5 pm throughout the day and is referred to as a fully shaded area.

Note: The abbreviations used in this table apply throughout the manuscript and will not be repeated thereafter.

**Figure 1:**
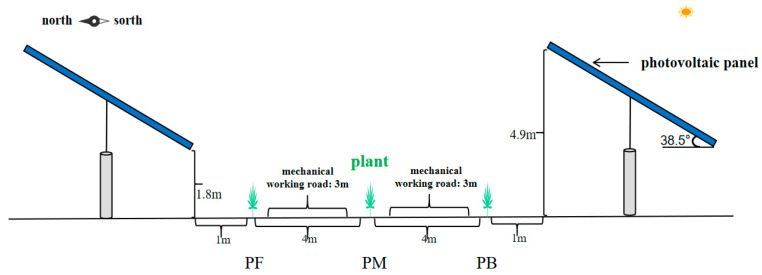
Schematic diagram of vegetation planting between photovoltaic panels.

#### 2.1.2. Plant Biomass Between Rows of Photovoltaic Panels

**Figure 2:**
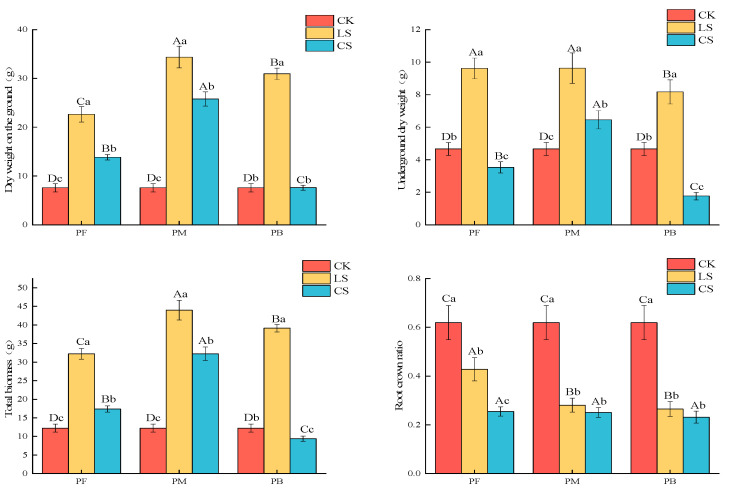
The impact of different ecological governance measures within the photovoltaic panel area on the biomass of *H. ammodendron*. Note: Different lowercase letters indicate significant differences in plant biomass indicators among ecological governance measures at the same location (*p* < 0.05). In contrast, different uppercase letters indicate significant differences in plant biomass indicators across different locations under the same ecological governance measure (*p* < 0.05).

Different sampling locations and ecological governance measures significantly influence the growth of *H. ammodendron*. The aboveground biomass of *H. ammodendron* (LS) and *H. ammodendron* (CS), in the PF and PM positions of the photovoltaic panel channel, was significantly higher than that of the CK (*p* < 0.05) (Figure 2). Notably, the LS aboveground biomass in the PM position of the photovoltaic panel channel in the photovoltaic panel area reached its maximum. The aboveground biomass of *H. ammodendron* (LS) within the photovoltaic panel area was in the following order: PM had the highest, followed by PB and then PF. Similarly, the aboveground biomass of *H. ammodendron* (CS) was the highest in the PM position, followed by the PF and then PB positions (Figure 1). The underground dry weight in the PF, PM, and PB positions of the photovoltaic panel area under LS ecological governance was significantly higher than that of the control group and CS ecological governance (*p* < 0.05). Furthermore, the underground dry weight in the middle of the photovoltaic panel area was in the following order: LS was the governance measure with the highest value, followed by the CS governance measure, and the CK had the lowest value. The total biomass of *Haloxylon ammodendron* under the LS ecological governance measure was significantly greater than that of both the control and CS groups (*p* < 0.05). And in the same mechanical working road, the total biomass of PF and PM *Haloxylon ammodendron* under the two ecological governance measures was significantly greater than that of the CK (*p* < 0.05). The root-to-shoot ratio of the CK was significantly greater than that of the LS and CS ecological governance measures (*p* < 0.05). The root-to-shoot ratio of *H*. *ammodendron* under the LS ecological governance measure was significantly greater than that under the CS ecological governance measure (*p* < 0.05). Furthermore, the root-to-shoot ratio under the LS ecological governance measure was significantly higher than that under the CS ecological governance measure (*p* < 0.05).

#### 2.1.3. Correlation Between Plant Growth and Biomass Within Photovoltaic Panel Areas

**Figure 3:**
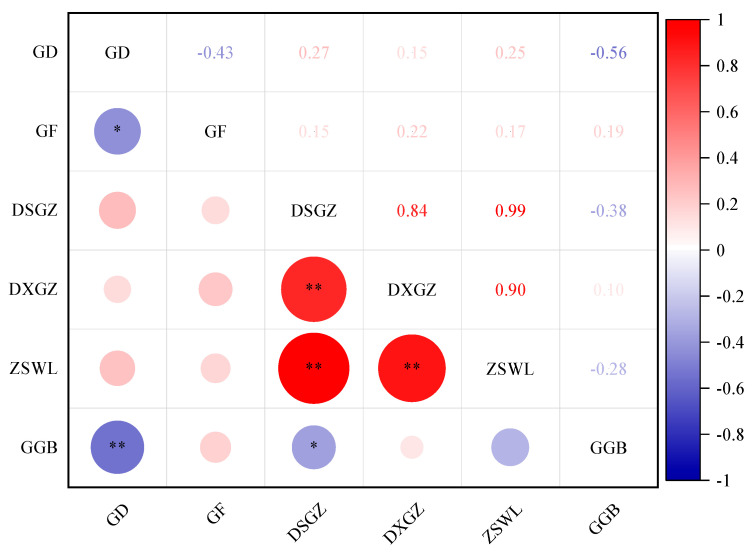
Correlation between growth and biomass of *H. ammodendron.* Note: GD represents height, GF represents crown width, DSGZ represents aboveground dry weight, DXGZ represents underground dry weight, ZSWL represents total biomass, GGB represents root-to-shoot ratio, * represents *p* < 0.05, and ** represents *p* < 0.01.

It is evident that the growth and biomass of *H. ammodendron* in the inter-row areas of photovoltaic panels exhibit varying degrees of correlation. There is a significant negative correlation between crown width and plant height (*p* < 0.05). Additionally, a highly significant negative correlation is observed between plant height and the root-to-shoot ratio (*p* < 0.01). Aboveground dry weight shows a highly significant positive correlation with underground dry weight and total biomass (*p* < 0.01) but a significant negative correlation with the root-to-shoot ratio (*p* < 0.05). Furthermore, underground dry weight is highly positively correlated with total biomass (*p* < 0.01) (Figure 3).

### 2.2. Characteristics of Changes in Soil Chemical Properties Under Different Ecological Governance Measures Within Photovoltaic Panel Areas

#### 2.2.1. Changes in Soil Nutrients Within Photovoltaic Panel Areas

In the 0–2 cm soil layer, organic matter and total phosphorus contents in LS-PF soil were significantly higher than those in the CK and CS-PF (*p* < 0.05) (Table 3). The available phosphorus and potassium contents in CS-PF soil were significantly higher than those in the CK and LS-PF (*p* < 0.05). In the 2–10 cm soil layer, the organic matter, total nitrogen, and available nitrogen contents in LS-PF soil were significantly higher than those in the CK and CS-PF soil (*p* < 0.05). The total potassium and available potassium contents in CS-PF soil were significantly higher than those in the CK and LS-PF (*p* < 0.05). At the 10–20 cm depth, the organic matter, total nitrogen, and available potassium contents in CS-PF soil were significantly higher than those in the CK and LS-PF (*p* < 0.05). At the 20–40 cm depth, the organic matter and total nitrogen contents in LS-PF soil were significantly higher than those in the CK and CS-PF (*p* < 0.05). The available nitrogen, available phosphorus, and available potassium contents in CS-PF soil were significantly higher than those in the CK and LS-PF (*p* < 0.05). The organic matter and total nitrogen contents in LS-PM soil at the 0–2 cm depth were significantly higher than those in the CK and CS-PM (*p* < 0.05). At the 2–20 cm depth, the organic matter content in CK soil was significantly higher than that in LS-PM and CS-PM (*p* < 0.05). At the 20–40 cm depth, the organic matter and total nitrogen contents in CK soil were significantly higher than those in LS-PM and CS-PM (*p* < 0.05). The total potassium and available nitrogen contents in LS-PM soil were significantly higher than those in the CK and CS-PM (*p* < 0.05). At the 0–2 cm depth, the total nitrogen, total potassium, and available nitrogen contents in LS-PB soil were significantly higher than those in the CK and CS-PB. At the 10–20 cm depth, the organic matter, total nitrogen, and total potassium contents in CK soil were significantly higher than those in LS-PB and CS-PB (*p* < 0.05).

A two-factor analysis of the different positions (PF, PM, and PB) and ecological governance measures (LS and CS) revealed that the ecological governance measures significantly affect soil organic matter, total nitrogen, and total phosphorus contents. Additionally, different locations significantly influence the contents of total phosphorus, total potassium, available nitrogen, available phosphorus, and available potassium in the soil. Furthermore, the interaction between location and ecological governance measures has a significant impact on soil organic matter content, total phosphorus content, available potassium content, and available phosphorus content.

#### 2.2.2. Correlation of Soil Nutrients Between Rows of Photovoltaic Panels

**Figure 4:**
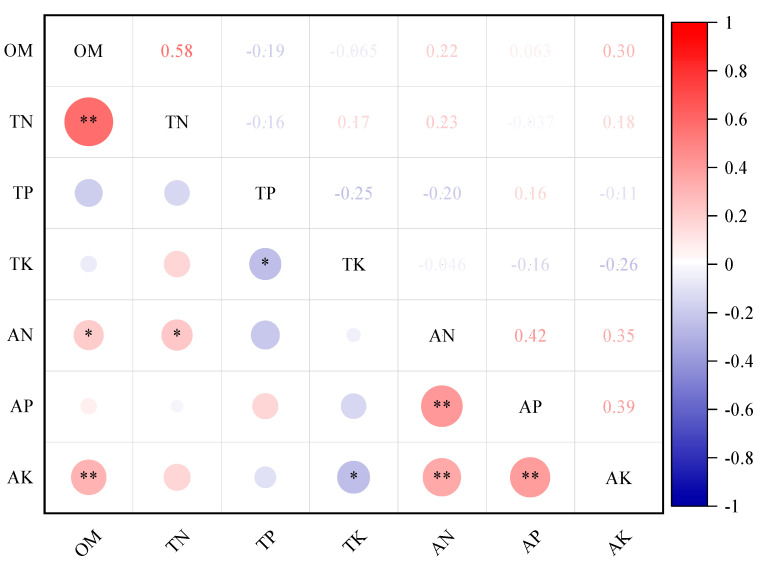
Correlation of soil nutrients between rows of photovoltaic panels. Note: OM represents soil organic matter, TN represents soil total nitrogen, TP represents soil total phosphorus, TK represents soil total potassium, AN represents soil available nitrogen, AP represents soil available phosphorus, and AK represents soil available potassium. * indicates *p* < 0.05, and ** indicates *p* < 0.01.

To investigate the changes in soil nutrients between rows of photovoltaic panels, this study conducted a correlation analysis of each soil nutrient indicator. Soil organic matter is significantly positively correlated with total nitrogen and available potassium (*p* < 0.01) and is also significantly positively correlated with available nitrogen (*p* < 0.05). A significant positive correlation is observed between soil total nitrogen and available nitrogen (*p* < 0.05). Conversely, total potassium, total phosphorus, and available potassium in the soil exhibit a significant negative correlation (*p* < 0.05). Furthermore, a highly significant positive correlation (*p* < 0.01) is identified among soil available nitrogen, available phosphorus, and available potassium (Figure 4).

### 2.3. Response of Plant Growth Between Rows of Photovoltaic Panels to Environmental Factors

#### 2.3.1. Analysis of Plant Growth and Soil Nutrient Redundancy Under Different Ecological Governance Measures for Photovoltaic Panels

**Figure 5:**
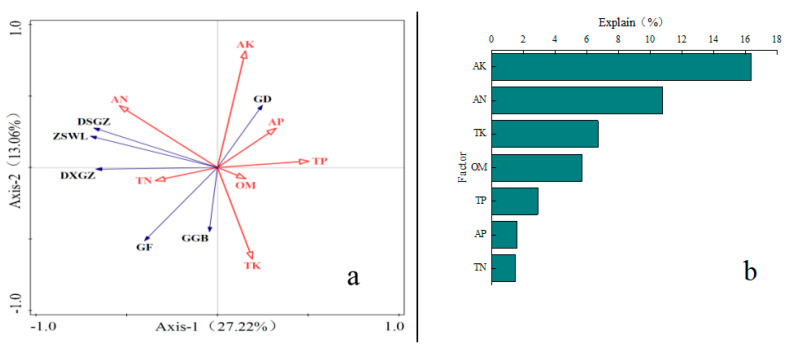
Redundancy analysis (RDA) of growth indicators of *H. ammodendron* and soil nutrients. Note: (**a**) represents the redundancy analysis ranking of plant traits and soil nutrients, (**b**) represents the results by redundancy analysis ordination with the first two axes and Monte Carlo permutation test. GD represents height, GF represents crown width, DSGZ represents aboveground dry weight, DXGZ represents underground dry weight, ZSWL represents total biomass, GGB represents root-to-shoot ratio, AN represents soil available nitrogen, AK represents soil available potassium, AP represents soil available phosphorus, OM represents soil organic matter, TP represents soil total phosphorus, TK represents soil total potassium, and TN represents soil total nitrogen.

To explore the relationship between plants growth and soil nutrient factors, a redundancy analysis (RDA) was performed. As shown in Figure 5a, the total interpretation rate of soil nutrients reached 35.83%, with the first and second axes of soil nutrients explaining 27.22% and 13.06% of the variation in shrub growth indices. Figure 5b ranks the contents of soil available potassium (AK), soil available phosphorus (AP), soil available nitrogen (AN), soil organic matter (OM), soil total phosphorus (TP), soil total potassium (TK), and soil total nitrogen (TN). The influence (explanatory power) of soil nutrient factors on the growth of *H. ammodendron* is ranked as follows: AK > AN > TK > OM > TP > AP > TN. Among these, AK (F = 7.2, *p* < 0.01) is identified as the key factor influencing the growth indicators of *H. ammodendron*, with an interpretation rate of 16.4%. The first axis primarily reflects the influence of soil organic matter, soil total nitrogen, and soil total potassium contents on the growth of *H. ammodendron*, while the second axis mainly reflects the effect of soil available nitrogen content. The angle between soil available nitrogen and growth indicators such as aboveground dry weight, underground dry weight, total biomass, and crown width is less than 90°, indicating that these growth indicators increase as soil available nitrogen content increases. Similarly, the angle between soil total potassium content and growth indicators such as crown width and root-to-crown ratio is less than 90°, suggesting that these indicators increase with the rise in soil total potassium content. Conversely, the angle between soil available potassium content and growth indicators such as total biomass, underground dry weight, crown width, and root-to-shoot ratio is greater than 90°, indicating a significant negative correlation. Therefore, soil available nitrogen and soil available potassium are critical factors influencing the growth of *H. ammodendron*.

#### 2.3.2. Principal Component Analysis of Plant Growth and Soil Nutrients Under Different Ecological Governance Measures for Photovoltaic Panels

The correlation between different indicators is significant, demonstrating strong connections among them. Consequently, principal component analysis (PCA) was employed for data analysis. PCA was performed on 13 indicators, including the growth parameters of *H. ammodendron* and soil nutrients. Principal component eigenvalues and contribution rates were used as criteria for selecting the principal components [23]. Based on the criterion of eigenvalues greater than 1, five principal components were extracted, accounting for a cumulative variance contribution rate of 84.34% (Table 4). The principal component loadings of each indicator, representing the correlation coefficients between each indicator and the relevant principal components, are shown in the table below. The first principal component explains 28.79% of the original information. *H. ammodendron* indicators such as aboveground dry weight, underground dry weight, total biomass, and soil available nitrogen exhibit large positive coefficient values, indicating that higher values of the first principal component correspond to higher values of these four indicators. The second principal component explains 21.42% of the original information, with significant positive coefficient values for *H. ammodendron* height, soil available phosphorus, and soil available potassium and significant negative coefficient values for *H. ammodendron* crown width.

This indicates that higher values of the second principal component correspond to greater *H. ammodendron* height, soil available phosphorus, and potassium content but smaller *H. ammodendron* crown width. The third principal component explains 14.45% of the original information and has significant positive coefficient values for soil organic matter and total nitrogen, indicating that higher values of the third principal component correspond to higher soil organic matter and total nitrogen content. The fourth principal component explains 10.56% of the original information, with soil available phosphorus showing a large negative coefficient value, indicating that higher values of the fourth principal component correspond to lower soil available phosphorus content. Finally, the fifth principal component explains 9.11% of the original information and has a large positive coefficient value for total phosphorus, indicating that higher values of the fifth principal component correspond to higher soil total phosphorus content.

#### 2.3.3. Gray Correlation Analysis of Plant Growth and Soil Nutrients Under Different Ecological Governance Measures for Photovoltaic Panels

To further explore the impact of various ecological management strategies for photovoltaic panels on plant growth and soil physicochemical properties, this study employed the gray relational analysis method for a comprehensive evaluation. As shown in Table 5, the ranking of the associated values is as follows: LS-PM > LS-PF > LS-PB > CS-PM > CS-PF > CK > CS-PB. Among these, LS-PM exhibits the most significant improvement in plant growth and soil conditions, followed by LS-PF, whereas CS-PB demonstrates the least favorable effects.

## 3. Discussion

### 3.1. Effects of Different Ecological Governance Measures and Sampling Locations on the Growth of H. ammodendron

The layout of photovoltaic brackets alters the underlying surface structure, increasing surface roughness and reducing wind speed. These changes contribute to wind prevention, sand fixation, and an increase in air and soil temperature and humidity levels [24]. This study demonstrated that the plant height of *H. ammodendron* at the front, middle, and back positions under two ecological governance measures was significantly greater than that of the control. These findings align with the results of Du et al. [25], suggesting that photovoltaic panels provide a shading effect, which reduces water evaporation and enhances the vegetation growth environment [26]. Observations from 8:00 am to 5:00 pm revealed variations in sunlight intensity at different positions between the photovoltaic panels and the roadway. The light intensity was in the following order: CK received the most abundant sunlight, followed by PF (fully sunny) and then PM (partially shaded and partially sunny), while PB did not receive sunlight throughout the day (fully shaded). The results of this study revealed that the plant height of *H. ammodendron* in fully sunny areas was greater than in fully shaded areas. In contrast, previous studies have shown that increasing levels of shading lead to greater leaf length, leaf width, and plant height in *Petasites japonicus* [27]. The leaf length and plant height of ground cover plants, such as *Potentilla chinensis*, *Oxalis corymbosa*, and *Iris lactea*, gradually decrease with the increase in the level of shading. This variation arises because each plant responds differently to light conditions. Under shading conditions, plants adapt through either shade avoidance or shade tolerance mechanisms [28]. The shade avoidance response is primarily characterized by tissue elongation, and most plants exhibit this response under low-light conditions. In contrast, plants with shade tolerance display a weaker tissue elongation response, but their physiological and biochemical indicators undergo significant changes. Shade avoidance and shade tolerance are not mutually exclusive mechanisms: they often occur simultaneously in most plants, but their dominance varies. Plants with strong shade tolerance exhibit a milder shade avoidance response, minimal changes in morphology, and relatively stable physiological processes [29,30]. This study found that both ecological governance measure and location significantly affected the growth of *H. ammodendron*. Additionally, the interaction between location and ecological governance measure also had a significant impact on its growth. Under varying environmental conditions, the cellular structure, physiological processes, and biochemical properties of plants undergo corresponding changes to adapt to their growth environment [31]. Compared with fully shaded branches, the growth rate and mortality rate of leaves on branches of *Salix matsudana* exposed to direct sunlight are higher than those on branches without shade and those in shade. Shade treatment significantly affected the plant’s net photosynthetic rate and nocturnal respiration rate. In terms of biomass allocation, branches exposed to direct sunlight exhibit higher branch biomass, greater total branch length, increased biomass of branches and leaves, and a larger branch-to-leaf weight ratio compared with unshaded branches. Shade can reduce evapotranspiration and help retain moisture, thereby reducing water loss [32,33]. This study found that aboveground biomass under the two ecological governance measures, both in front of and beneath the photovoltaic panels, was significantly higher than that of the control. This result may be attributed to the ability of photovoltaic panels to collect rainwater, increase soil temperature and humidity, and maintain adequate water supply for the normal growth of *H. ammodendron* [34]. Research has shown that both the direct planting of *H. ammodendron* and its planting after laying straw checkerboard barriers resulted in a significantly lower root-to-shoot ratio compared with the control. This may represent a physiological adaptation mechanism of *H. ammodendron* to arid environments, enabling it to extract water from deeper soil layers. This finding is consistent with results observed in *Robinia pseudoacacia*, which adapts to water-deficient environments by increasing root depth to compensate for soil moisture deficits [35]. Wang Yayun’s study on the water transport and carbon metabolism characteristics of *H. ammodendron* and *Alhagi camelorum* under drought stress reached a similar conclusion [36]. Under ecological governance measures where plants are directly planted in bare sand, the crown width, aboveground biomass, underground biomass, and total biomass of *H. ammodendron* were significantly greater compared with those of plants grown after the establishment of straw checkerboard barriers. This difference may result from the ability of straw checkerboard barriers to retain windborne seeds, thereby enhancing plant diversity in the area. The increased competition among species likely suppressed the growth of *H. ammodendron*, leading to its slightly weaker performance.

### 3.2. Effects of Different Ecological Governance Measures and Sampling Locations on Soil Nutrients Under H. ammodendron

The restoration and maintenance of soil nutrients are critical indicators of functional recovery and self-sustainability in terrestrial degraded ecosystems. Consequently, soil physical and chemical properties are widely recognized as essential metrics for evaluating ecosystem restoration [37,38]. In this study, the two ecological governance measures significantly influenced soil nutrient indexes. When the soil layer depth was 0–2 cm, the organic matter content in PF and PB under LS treatment, as well as PB under CS treatment, increased significantly. Additionally, the total nitrogen content in PM soil under LS treatment and the total phosphorus content in PF soil under LS treatment both increased significantly. It is possible that after the implementation of ecological governance between the mechanical working road and adjacent photovoltaic panels, the roots of vegetation, dead branches and leaves, and plant secretions continue to accumulate, leading to increased nutrient input and the accumulation of soil organic matter. This, in turn, enhances microbial activity and the decomposition rate of soil organic matter, ultimately resulting in the accumulation of soil nutrients [39], which is consistent with the results of Li Yuzhang’s research [40]. In this study, the available phosphorus and potassium contents in PF soil were higher than those under the LS and CS governance measures. The straw checkerboard barriers reduced wind erosion, enhanced the interception of airborne sand and dust, and subsequently increased the nutrient content in CS soil. The organic matter content and total phosphorus content in the surface layer of PF were higher than those in PB under the LS governance measure. This may be attributed to the front eaves of the photovoltaic panels, which increased soil moisture through water collection without obstructing sunlight. These favorable hydrothermal conditions enhanced soil microbial activity, promoting the decomposition of plant residues and significantly increasing soil organic matter content [41,42]. However, the total nitrogen, available nitrogen, and available phosphorus contents in the soil in front of the photovoltaic panel were lower than those behind the panel. This conclusion differs from Li Wenlong’s [43] findings on the impact of vegetation restoration measures on soil nutrients between photovoltaic arrays. This discrepancy may be attributed to variations in water and heat conditions in the study area, as well as differences in the plant species studied. Additionally, the planting period of plants can influence the soil improvement effect beneath photovoltaic panels. Wu Zhiquan [44] found that after cultivating *Euryops pectinatus* in a photovoltaic park for one year, the total nutrient content of the soil beneath the panels significantly increased. Moreover, with the increase in the number of planting years, the impact of plants on soil improvement between photovoltaic panels becomes more pronounced [45]. The surface soil in the sandy area exhibited high porosity, allowing soil moisture to infiltrate easily. Additionally, the total nitrogen and total potassium contents in the deep layer (2–40 cm) of LS soil in front of the photovoltaic panels were significantly higher than those in the surface layer (0–2 cm).

### 3.3. Comprehensive Analysis

It is challenging to naturally restore the ecological environment of photovoltaic power plants constructed in deserts, arid regions, and the Gobi Desert after the disturbance and damage caused by engineering activities [46]. Comprehensive sand fixation and protection measures, including planting vegetation and establishing straw checkerboard barriers, can gradually stabilize the sand surface and create favorable conditions for plant settlement [47]. This has been thoroughly validated in the present study. In this study, the growth and biomass of *H. ammodendron* exhibited significant positive correlations with aboveground dry weight, underground dry weight, and total biomass (*p* < 0.01). Conversely, a significant negative correlation was observed between the height of *H. ammodendron* and the root-to-shoot ratio (*p* < 0.01). Furthermore, the analysis of soil nutrients under photovoltaic panels revealed that soil organic matter was significantly positively correlated with total nitrogen and available potassium (*p* < 0.01) and showed a significant positive correlation with available nitrogen (*p* < 0.05). A significant positive correlation was observed among soil available nitrogen, available phosphorus, and available potassium (*p* < 0.01). A principal component analysis of 13 indicators, including growth, biomass, and soil nutrients, revealed that the aboveground dry weight of *H. ammodendron* was the most representative metric for assessing its growth at different locations under various ecological governance measures. A redundancy analysis of the growth, biomass, and soil nutrients of *H. ammodendron* identified soil available potassium as the key factor influencing its growth. Through a gray correlation analysis of plant growth and soil chemical properties, it was found that planting *Haloxylon ammodendron* on bare sandy land is more effective than planting it after installing grass grid sand barriers. This phenomenon may be attributed to the relatively short duration of grass grid sand barrier installation. Studies indicate that laying grass grid sand barriers for only 1–2 years does not significantly enhance soil nutrients. However, over time, as the duration of grass grid installation increases, the atmospheric dust captured by the grids continues to accumulate and decompose, nitrogen-fixing plants and biological crusts gradually develop, and the diversity of settled biological species increases. These biological processes become more pronounced, accelerating soil nutrient accumulation [48].

In summary, different ecological governance measures implemented in a photovoltaic park and variations in positions influence the growth of *H. ammodendron* and soil nutrient dynamics. The effectiveness of ecological governance measures is determined by multiple factors, while changes in soil nutrients represent a long-term and continuous process. This study focuses on the short-term effects of two ecological governance measures on the growth of *H. ammodendron* and soil nutrients within a photovoltaic park, observed two years after the construction of the photovoltaic park. The long-term monitoring of soil nutrients, plant growth, and ecological impacts is required to better understand and optimize these governance measures.

## 4. Materials and Methods

### 4.1. Overview of the Research Area

The study area is located at the northeastern edge of the Ulan Buhe Desert, within Dengkou County, Bayannur City, Inner Mongolia. The region experiences an arid climate and harsh environmental conditions, with an average annual temperature of 7.6 °C, an average wind speed of 3.4 m/s, and annual evaporation of 2327 mm. Winters and springs are characterized by drought and minimal rainfall. The average temperature in January is −8.9 °C, with an extreme minimum temperature of −30.8 °C. The area is dominated by prevailing northwesterly winds, leading to significant sand damage and frequent windy days, which average 12.5 days per year. These conditions have a substantial adverse impact on both production and daily life. Southeastern winds are more prevalent during the summer and autumn seasons, which are also the periods of concentrated precipitation. The average annual precipitation is 145 mm, contributing approximately 60–80% of the total annual rainfall. The average temperature in July is 21.8 °C, with an extreme maximum temperature of 41.1 °C. The research area receives abundant sunshine and has an extended growing season, with an annual sunshine duration exceeding 3210 h and a frost-free period of approximately 136 days. During the plant growth period, the accumulated temperature is approximately 3100 °C, and the day–night temperature difference is 14.5 °C, creating favorable conditions for plant growth [49].

**Figure 6:**
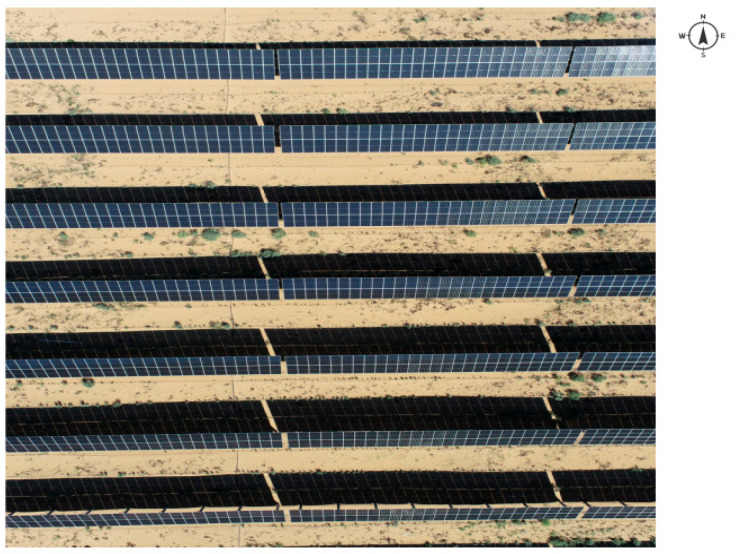
On-site photo of photovoltaic power station.

The 100 MW photovoltaic sand control project, a collaboration between State Power Investment Group and Yishite Group, is located in Dengkou County, Inner Mongolia, with coordinates 106°53′39″ E and 40°23′20″ N. The project is situated approximately 20 km from the county seat. The construction of the power station began in October 2021, and it became operational in November 2022. The layout and specifications of the photovoltaic panels in the research area are as follows: The photovoltaic panels face the south and are arranged in an overall east–west direction, with a distance of 12 m between adjacent rows of photovoltaic panels. The vertical height of the front eaves of the photovoltaic panels from the ground is 1.8 m, while the vertical height of the rear eaves is 4.9 m. The overall specification of a single set of photovoltaic panels is 1.1 m × 1.1 m × 4 pieces × 24 pieces, and the photovoltaic panel inclination angle is 38.5° (Figure 6).

In March 2023, ecological governance was implemented at the photovoltaic power station following the “1441” model: *Haloxylon ammodendron* was planted 1 m from the front eaves of the photovoltaic panel. *Haloxylon ammodendron* was planted 1 m from the rear eaves of adjacent photovoltaic panels. *Haloxylon ammodendron* was planted in the center of the mechanical working road between two adjacent photovoltaic panels. The plant spacing between *Haloxylon ammodendron* plants was 1 m, and the row spacing was 4 m (Figure 1). Two types of ecological governance measures were implemented: planting *Haloxylon ammodendron* directly on exposed sandy land and planting it after laying straw checkerboard barriers. Both methods were managed by using drip irrigation technology following planting. This study focuses on two ecological governance measures: the direct planting of *Haloxylon ammodendron* on bare sandy land and its planting after laying straw checkerboard barriers on the same land. *Haloxylon ammodendron* planted on bare sandy land at the same time as the research group served as the control group.

### 4.2. Research Methods

#### 4.2.1. Field Investigation and Sampling

In May 2024, for the two ecological governance measures selected in this study, five lanes between LS photovoltaic panels and five lanes between CS photovoltaic panels were randomly chosen. A 10 m long transect line was established at the PF, PM, and PB positions, and the height and crown width of *H. ammodendron* were measured along the transect. Additionally, one *H. ammodendron* plant was randomly excavated from each transect. The aboveground and underground parts of each plant were separated, and the roots were cleaned to remove any attached soil. The *H. ammodendron* sample was then placed in an envelope bag and transported to the laboratory. A soil drill was used to extract soil near the area where the *H. ammodendron* was excavated at the PF, PM, and PB positions. The soil samples were collected at four depths: 0–2 cm, 2–10 cm, 10–20 cm, and 20–40 cm. The control group consisted of *H. ammodendron* plants cultivated on bare sand dunes under ecological management (CK) during the same period.

#### 4.2.2. Sample Processing and Measurement

The underground parts of the *H. ammodendron* collected in the wild were rinsed with water and wiped to remove impurities that could adhere to the roots and affect the test results. Both the aboveground and underground parts of *H. ammodendron* were dried in a drying oven at 65 °C until constant weight. The dry weight was then measured to determine the aboveground and underground biomass.

The total biomass was calculated as the sum of aboveground biomass and underground biomass.

The root-to-shoot ratio was calculated as the underground biomass divided by the aboveground biomass.

The soil sample was sieved through a 2 mm mesh to remove visible roots, straw residues, and gravel and then air-dried. After air-drying, the soil sample was further sieved through 1 mm and 0.25 mm meshes for the determination of soil nutrient indicators. The specific methods used for determining soil nutrient indicators are provided in Table 6.

### 4.3. Data Processing

This experiment utilized Microsoft Excel 2010 and SPSS 25.0 for data processing and statistical analysis, while Origin 2024 was used for plotting. A one-way ANOVA was conducted to test the effects of different photovoltaic panel locations at the same soil depth, as well as the effects of different soil depths at the same sampling location, on soil OM, TN, TP, TK, AN, AP, and AK. A two-way ANOVA was employed to evaluate the effects of different ecological management measures (treatments) and sampling locations (PF, PM, and PB) on soil OM, TN, TP, TK, AN, AP, and AK. The LSD multiple comparison method was applied to test the significance of differences. Additionally, Canoco 5 was used to analyze the relationship between the growth of *H. ammodendron* and soil nutrients under different ecological management measures in the photovoltaic panel roadways.

## 5. Conclusions

The growth, biomass, and soil nutrient dynamics of *H. ammodendron* are significantly influenced by both the ecological governance measures implemented within photovoltaic power plants and the positioning of photovoltaic panels. This study reveals that planting *H. ammodendron* directly in bare sandy soil leads to significantly greater crown width, aboveground biomass, belowground biomass, and total biomass compared to planting after the installation of straw checkerboard barriers. However, the ecological governance measure of planting *H. ammodendron* with straw checkerboard barriers resulted in higher soil total potassium, available phosphorus and available potassium contents in the areas located in front of the photovoltaic panels compared with direct planting in bare sandy soil.

## Figures and Tables

**Table 2:** The impact of different ecological governance measures for photovoltaic panels on the growth of *H. ammodendron*.

Treatment	Position	Height (cm)	Crown Width (cm)
CK	PF	34.00 ± 2.31 Dc	30.90 ± 2.19 Da
	PM	34.00 ± 2.31 Dc	30.90 ± 2.19 Da
	PB	34.00 ± 2.31 Dc	30.90 ± 2.19 Db
LS	PF	50.36 ± 2.69 Ab	29.82 ± 0.99 Cb
	PM	42.32 ± 1.57 Cb	31.80 ± 1.46 Ba
	PB	45.08 ± 2.90 Bb	34.30 ± 1.60 Aa
CS	PF	48.44 ± 1.89 Ba	29.40 ± 1.45 Ab
	PM	51.08 ± 3.44 Aa	24.32 ± 1.61 Bb
	PB	47.40 ± 2.83 Ba	29.70 ± 1.54 Ac
Position		**	**
Treatment		**	**
Position * Treatment		**	**

Note: Different lowercase letters indicate significant differences (*p* < 0.05) in plant growth indicators between different ecological governance measures at the same location, while different uppercase letters indicate significant differences (*p* < 0.05) in plant growth indicators for the same ecological governance measure at different locations. ** indicates a highly significant difference. Same below.

**Table 3:** The impact of different ecological governance measures and the spatial arrangement of photovoltaic panels on soil nutrients.

Position	Treatment	Soil Depth (cm)	Organic Matter (g/kg)	Total Nitrogen (g/kg)	Total Phosphorus (g/kg)	Total Potassium (g/kg)	Available Nitrogen (mg/kg)	Available Phosphorus (mg/kg)	Available Potassium (mg/kg)
PF	CK	0–2	3.26 ± 0.25 Ba	0.17 ± 0.01 Ab	0.18 ± 0.01 Ba	22.44 ± 0.39 Aa	20.49 ± 0.49 ABa	2.74 ± 0.16 Ba	181.01 ± 0.49 Cb
		2–10	2.50 ± 0.13 Bb	0.19 ± 0.01 Bab	0.17 ± 0.01 Aa	21.92 ± 0.82 Ba	18.63 ± 0.82 Bb	2.55 ± 0.10 Aab	181.13 ± 1.23 Bb
		10–20	3.18 ± 0.16 Ba	0.20 ± 0.01 Ba	0.18 ± 0.02 Aa	21.83 ± 0.71 Aa	16.07 ± 0.50 Bc	2.21 ± 0.16 ABc	182.80 ± 1.51 Bb
		20–40	2.66 ± 0.09 Bb	0.18 ± 0.02 Bab	0.17 ± 0.01 Aa	20.56 ± 0.62 Ab	14.50 ± 0.82 Cd	2.29 ± 0.18 Bbc	185.18 ± 1.27 Ba
	LS	0–2	3.85 ± 0.11 Aa	0.13 ± 0.02 Bc	0.21 ± 0.01 Aa	17.90 ± 0.67 Bc	21.71 ± 0.93 Aa	2.34 ± 0.05 Ca	192.07 ± 2.00 Ba
		2–10	3.48 ± 0.30 Ab	0.25 ± 0.01 Aa	0.16 ± 0.03 Ab	20.46 ± 1.19 Bb	20.78 ± 1.04 Aa	2.38 ± 0.66 Aa	181.23 ± 1.58 Bbc
		10–20	2.80 ± 0.09 Cc	0.21 ± 0.01 Bb	0.17 ± 0.01 Ab	26.22 ± 1.01 Ba	18.39 ± 1.87 ABb	2.03 ± 0.14 Ba	178.77 ± 2.12 Cc
		20–40	3.09 ± 0.11 Ac	0.23 ± 0.02 Aab	0.19 ± 0.02 Aab	20.80 ± 1.43 Ab	17.54 ± 0.14 Bb	2.18 ± 0.10 Ba	183.60 ± 0.66 Bb
	CS	0–2	3.04 ± 0.13 Bb	0.19 ± 0.01 Ab	0.16 ± 0.01 Ba	22.72 ± 0.49 Ab	20.22 ± 0.59 Bb	3.94 ± 0.14 Aa	209.60 ± 0.9 A0 a
		2–10	2.63 ± 0.04 Bc	0.16 ± 0.02 Bc	0.16 ± 0.12 Aa	26.63 ± 0.77 Aa	12.49 ± 0.13 Cc	2.53 ± 0.13 Abc	202.63 ± 0.81 Ab
		10–20	3.97 ± 0.11 Aa	0.26 ± 0.01 Aa	0.19 ± 0.01 Aa	22.26 ± 0.42 Ab	19.32 ± 0.81 Ab	2.46 ± 0.12 Ac	203.00 ± 0.20 Ab
		20–40	2.57 ± 0.12 Bc	0.19 ± 0.01 Bb	0.13 ± 0.01 Ba	16.53 ± 0.89 Bc	23.35 ± 0.54 Aa	2.75 ± 0.08 Ab	208.47 ± 0.84 Aa
PM	CK	0–2	3.26 ± 0.25 Ba	0.17 ± 0.01 Bb	0.18 ± 0.01 Aa	22.44 ± 0.39 Aa	20.49 ± 0.49 Ba	2.74 ± 0.16 Ba	181.01 ± 0.49 Cb
		2–10	2.50 ± 0.13 Ab	0.19 ± 0.01 Aab	0.17 ± 0.01 Aa	21.92 ± 0.82 Aa	18.63 ± 0.82 Bb	2.55 ± 0.10 Aab	181.13 ± 1.23 Bb
		10–20	3.18 ± 0.16 Aa	0.20 ± 0.01 Aa	0.18 ± 0.02 Ba	21.83 ± 0.71 Ba	16.07 ± 0.50 Ac	2.21 ± 0.16 Ac	182.80 ± 1.51 Cb
		20–40	2.66 ± 0.09 Ab	0.18 ± 0.02 Aab	0.17 ± 0.01 Ba	20.56 ± 0.62 Bb	14.50 ± 0.82 Bd	2.29 ± 0.18 Bbc	185.18 ± 1.27 Aa
	LS	0–2	3.77 ± 0.10 Aa	0.26 ± 0.01 Aa	0.17 ± 0.03 Aa	17.61 ± 0.78 Ba	32.09 ± 1.22 Ab	2.59 ± 0.23 Bb	223.73 ± 1.86 Ba
		2–10	1.62 ± 0.07 Cc	0.12 ± 0.03 Ab	0.13 ± 0.01 Cb	17.98 ± 0.89 Ba	11.90 ± 0.80 Cc	2.49 ± 0.14 Ab	164.13 ± 2.87 Cd
		10–20	2.11 ± 0.07 Cb	0.14 ± 0.02 Bb	0.17 ± 0.01 Ba	26.77 ± 0.99 Ac	8.12 ± 0.55 Cd	1.74 ± 0.06 Bc	188.53 ± 4.22 Bb
		20–40	1.99 ± 0.06 Bb	0.15 ± 0.01 Bb	0.19 ± 0.01 Ba	23.94 ± 0.70 Ab	37.85 ± 1.09 Aa	3.14 ± 0.14 Aa	179.63 ± 1.85 Bc
	CS	0–2	2.83 ± 0.15 Ca	0.18 ± 0.01 Ba	0.19 ± 0.01 Ab	16.60 ± 0.46 Ba	30.65 ± 0.95 Aa	3.46 ± 0.09 Aa	314.23 ± 5.75 Aa
		2–10	2.17 ± 0.08 Bc	0.14 ± 0.05 Aa	0.15 ± 0.01 Bb	21.65 ± 0.64 Ab	21.65 ± 1.13 Ab	2.47 ± 0.08 Ac	203.17 ± 1.53 Ac
		10–20	2.47 ± 0.10 Bb	0.18 ± 0.01 Aa	0.21 ± 0.01 Ab	17.20 ± 1.09 Ca	10.77 ± 0.45 Bc	1.78 ± 0.12 Bd	224.20 ± 1.54 Ab
		20–40	1.97 ± 0.11 Bc	0.15 ± 0.01 Ba	0.68 ± 0.07 Aa	16.21 ± 0.23 Ca	12.22 ± 1.81 Bc	3.08 ± 0.13 Ab	180.67 ± 1.22 Bd
PB	CK	0–2	3.26 ± 0.25 Aa	0.17 ± 0.01 Bb	0.18 ± 0.01 Aa	22.44 ± 0.39 Ba	20.49 ± 0.49 Ba	2.74 ± 0.16 Aa	181.01 ± 0.49 Cb
		2–10	2.50 ± 0.13 Ab	0.19 ± 0.01 Aab	0.17 ± 0.01 Ba	21.92 ± 0.82 Aa	18.63 ± 0.82 ABb	2.55 ± 0.10 Aab	181.13 ± 1.23 Bb
		10–20	3.18 ± 0.16 Aa	0.20 ± 0.01 Aa	0.18 ± 0.02 Ba	21.83 ± 0.71 Aa	16.07 ± 0.50 Cc	2.21 ± 0.16 Bc	182.80 ± 1.51 Bb
		20–40	2.66 ± 0.09 Bb	0.18 ± 0.02 Aab	0.17 ± 0.01 Aa	20.56 ± 0.62 Ab	14.50 ± 0.82 Cd	2.29 ± 0.18 Abc	185.18 ± 1.27 Ba
	LS	0–2	2.52 ± 0.41 Bab	0.22 ± 0.01 Aa	0.16 ± 0.01 Bb	26.79 ± 1.10 Aa	27.28 ± 0.96 Aa	2.44 ± 0.06 Bb	218.47 ± 1.00 Ba
		2–10	2.25 ± 0.06 Bb	0.14 ± 0.01 Bc	0.16 ± 0.01 Bb	16.53 ± 2.35 Bc	20.96 ± 2.69 Ab	2.47 ± 0.10 Ab	200.23 ± 1.80 Ab
		10–20	2.47 ± 0.01 Cab	0.15 ± 0.01 Bc	0.18 ± 0.01 Ba	16.92 ± 0.59 Bc	18.64 ± 1.16 Bb	3.02 ± 0.20 Aa	196.37 ± 3.52 Ab
		20–40	2.71 ± 0.01 Ba	0.18 ± 0.02 Ab	0.13 ± 0.01 Bc	20.94 ± 1.69 Ab	20.26 ± 0.94 Ab	2.15 ± 0.06 Ac	197.77 ± 1.50 Ab
	CS	0–2	3.82 ± 0.07 Aa	0.18 ± 0.01 Ba	0.17 ± 0.01 ABc	17.88 ± 1.01 Cb	20.30 ± 0.89 Bb	2.86 ± 0.13 Aa	271.37 ± 1.60 Aa
		2–10	2.32 ± 0.11 Bd	0.17 ± 0.02 ABab	0.24 ± 0.01 Aa	21.65 ± 1.44 Aa	16.54 ± 1.06 Bc	2.16 ± 0.10 Bb	161.63 ± 1.21 Cd
		10–20	2.68 ± 0.03 Bc	0.15 ± 0.01 Bc	0.22 ± 0.01 Ab	17.92 ± 1.07 Bb	22.92 ± 1.01 Aa	1.94 ± 0.11 Bc	174.73 ± 0.80 Cc
		20–40	3.02 ± 0.07 Ab	0.15 ± 0.01 Bbc	0.19 ± 0.02 Ac	17.71 ± 1.00 Bb	16.65 ± 0.79 Bc	2.36 ± 0.09 Ab	195.67 ± 0.91 Ab
Treatment			**	*	*	ns	ns	ns	ns
Position			ns	ns	*	*	*	*	**
Treatment * Position			*	ns	*	ns	ns	*	*

Note: Lowercase letters indicate significant differences (*p* < 0.05) in soil nutrient indicators between different soil layers under the same treatment, while uppercase letters indicate significant differences (*p* < 0.05) in soil nutrient indicators within the same soil layer under different ecological management measures at the same location. * indicating significant differences, ** indicating a highly significant difference.

**Table 4:** Principal component analysis of plant growth and soil nutrients under different ecological management measures for photovoltaic panels.

Factor	Ingredient				
	1	2	3	4	5
Height	0.420	0.556	−0.397	0.159	0.367
Crown width	0.021	−0.673	0.449	−0.243	0.260
Aboveground dry weight	0.908	−0.328	−0.209	−0.049	0.066
Underground dry weight	0.757	−0.497	−0.031	0.331	−0.078
Total biomass	0.900	−0.376	−0.175	0.036	0.035
Root–crown ratio	−0.404	−0.367	0.433	0.446	−0.392
Organic matter	0.127	0.416	0.637	0.473	−0.001
Total nitrogen	0.372	0.014	0.754	−0.18	0.253
Total phosphorus	−0.351	0.349	0.014	0.471	0.622
Total potassium	−0.460	−0.360	0.117	−0.456	0.486
Available nitrogen	0.718	0.228	0.474	−0.041	0.067
Available phosphorus	0.089	0.578	0.188	−0.501	−0.351
Available potassium	0.429	0.763	0.101	−0.196	−0.045
Characteristic value	3.74	2.79	1.88	1.37	1.19
Contribution rate (%)	28.79	21.42	14.45	10.56	9.11
Accumulated contribution rate (%)	28.79	50.22	64.67	75.23	84.34

**Table 5:** Gray correlation analysis of plant growth and soil nutrients under different ecological management measures for photovoltaic panels.

Treatment	Associated Value	Ranking
CK	0.659	6
LS-PF	0.764	2
LS-PM	0.779	1
LS-PB	0.740	3
CS-PF	0.692	5
CS-PM	0.714	4
CS-PB	0.610	7

**Table 6:** Methods for determining soil nutrient indicators.

Measurement Indicators	Measurement Method	Reference
Soil organic matter, OM	Determined by the K_2_Cr_2_O_7_ external titration method	Soil Agrochemical Analysis [50]
Total nitrogen, TN	Measured by the semi-micro Kjeldahl method	
Total phosphorus, TP	Determined by using the NaOH fusion molybdenum antimony colorimetric method	
Total potassium, TK	Measured by the NaOH fusion flame photometry method	
Available nitrogen, AN	Determined by the alkali diffusion method	
Available phosphorus, AP	Measured by the NaHCO_3_ extraction colorimetric method	
Available potassium, AK	Determined by the NH_4_OAc extraction flame photometry method	

## Data Availability

The data presented in this study are available upon request to the corresponding author.
